# Complete mitochondrial genome of the bamboo snout beetle, *Cyrotrachelus buqueti* (Coleoptera: Curculionidae)

**DOI:** 10.1080/23802359.2017.1422411

**Published:** 2018-01-05

**Authors:** Wen-Jia Yang, Da-Xing Yang, Kang-Kang Xu, Yu Cao, Yong-Lu Meng, Yan Wu, Guo-Yong Li, Guo-Zhou Zhang, Ya-Wei Wang, Can Li

**Affiliations:** Guizhou Provincial Key Laboratory for Rare Animal and Economic Insects of the Mountainous Region, College of Biology and Environmental Engineering, Guiyang University, Guiyang, China

**Keywords:** *Cyrotrachelus buqueti*, bamboo snout beetle, mitochondrial genome

## Abstract

The bamboo snout beetle *Cyrotrachelus buqueti* (Coleoptera: Curculionidae) is a destructive forest pest and distributed widely in Southeast Asia. The 15,035 bp complete mitochondrial genome of the species consists of 13 protein-coding genes (PCGs), two ribosomal RNA genes (rRNAs), 21 transfer RNA genes (tRNAs) and a control region (GenBank accession no. MG674390). The *trnl* gene was not found in the *C. buqueti* mitogenome. The gene order and the orientation of *C. buqueti* were similar to those found in other Coleoptera species. The nucleotide composition was significantly biased (A, G, C, and T was 38.18%, 10.10%, 16.16%, and 35.56%, respectively) with A + T contents of 73.74%. ATG, ATA, ATT, AAT, and TTG were initiation codons and TAA, TAG, and T were termination codons. All the 21 tRNAs displayed a typical cloverleaf secondary structure, except for *trnS_1_* which lacked the dihydrouridine arm. Phylogenetic analysis was performed using 13 PCGs with 14 other beetles showed that *C. buqueti* is closely related to *Eucryptorhynchus brandti*, which agree with the traditional classification.

The bamboo snout beetle, *Cyrtotrachelus buqueti* Guerin-Meneville (Coleoptera: Curculionidae), is a highly destructive forest pest and distributed widely in Southeast Asia (Yang et al. 2017). The larvae of *C. buqueti* bore into the shoots of clumping bamboo species, causing serious damage to bamboo production (Yang et al. 2009). Adult specimens of *C. buqueti* were collected from Nanming District, Guiyang City, Guizhou Province, China (N26°33′, E106°46′). Samples have been deposited in the insect specimen room of Guiyang University with an accession number GYU-Col-20170001).

The circular mitochondrial genome of *C. buqueti* was 15,035 bp in length (GenBank accession no. MG674390) and included sets of genes, including 13 protein-coding genes (PCGs), two ribosomal RNA genes (*rrnL* and *rrnS*), 21 transfer RNA genes (tRNAs), and a large non-coding region (putative control region). The *trnl* was not found in the *C. buqueti* mitogenome, as observed in *Sympiezomias velatus* (Tang et al. 2017), another completely sequenced species in Coleoptera. The gene order and the orientation of *C. buqueti* were similar to those of putative ancestor of insects (Boore 1999). The nucleotide composition of was significantly biased (A, G, C, and T was 38.18%, 10.10%, 16.16%, and 35.56%, respectively) with A + T contents of 73.74%. The AT-skew and GC-skew of this genome were 0.036 and −0.231, respectively. Twenty-two genes were transcribed on the J-strand, whereas the others were oriented on the N-strand. Gene overlaps were present at eight gene junctions and involved a total of 20 bp; the longest overlap (7 bp) existed between *atp8* and *atp6*. A total of 59 bp intergenic spacers were found in 10 positions, ranging in size from 1 to 21 bp. The largest intergenic spacer sequence of 21 bp was located between *trnS_2_* and *nad1*. The control region was located between *rrnS* and *trnQ* gene with a length of 430 bp, and the A + T content was 81.16%.

All the 21 tRNAs were predicted to have typical cloverleaf secondary structures, except the gene *trnS_1_* (AGN) lacking a stable dihydrouridine arm, which were consistent with those reported in other animal mitogenomes (Wolstenholme 1992; Yuan et al. 2016). The length of these tRNAs ranged from 63 bp (*trnC* and *trnE*) to 71 bp (*trnK*), A + T content ranged from 58.46% (*trnN*) to 86.15% (*trnD*). The *rrnL* was located between *trnL_1_* and *trnV*, and *rrnS* resided between *trnV* and the control region. The *rrnL* was 1298 bp in length with A + T content of 78.43%, and the *rrnS* was 788 bp in length with A + T content of 75.13%.

The initial codons for 11 PCGs of *C. buqueti* were the canonical putative start codons ATN (ATG for *atp6*, *nad4L*, and *cob*; ATT for *cox2*, *atp8*, and *nad5*; ATA for *cox3*, *nad3*, *nad1*, and *nad4*; ATC for *nad2*). However, *cox1* and *nad6* used AAT and TTG as start codon, respectively. Nine PCGs were terminated with TAA or TAG, and the remaining PCGs including *cox1*, *cox3*, *nad4*, and *nad5* use a single T as stop codon. We analyzed the amino acid sequences of 13 PCGs with neighbour-joining method to construct the phylogenetic relationship of *C. buqueti* with 14 other representative bettles. The result showed that *C. buqueti* is closely related to *Eucryptorhynchus brandti* (Figure 1), which agree with the traditional classification.

**Figure 1.**
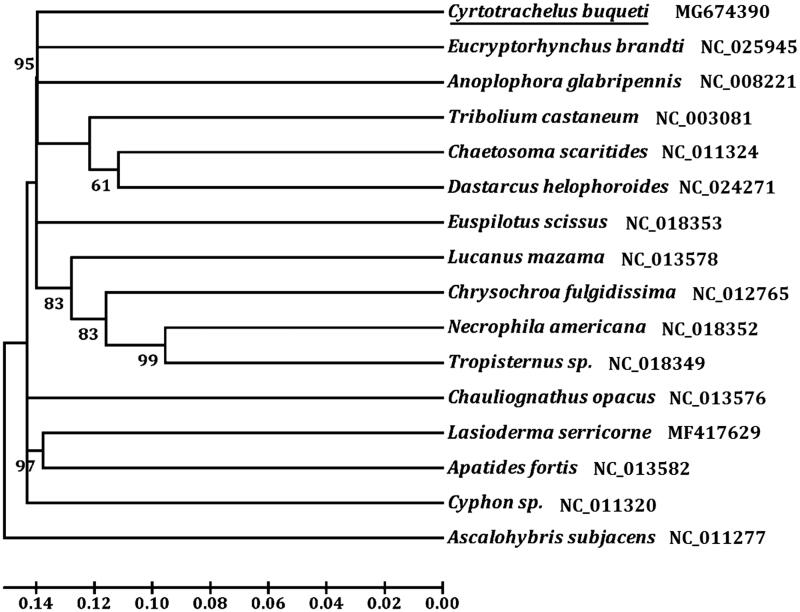
Phylogenetic tree showing the relationship between *C. buqueti* and 14 other beetles based on neighbour-joining method. *Ascalohybris subjacens* was used as an outgroup. GeneBank accession numbers of each species were listed in the tree.
